# Supplementary material for: Synthesis, Crystal Structure, Spectral Characterization and Antifungal Activity of Novel Phenolic Acid Triazole Derivatives
Source: Molecules. 2023 Oct 7;28(19):6970. doi: 10.3390/molecules28196970 (PMC10574244; doi:10.3390/molecules28196970)

Crystal structure determination

Table S1. Crystal and structure refinement data of **1a-3a** and **3b**

Complex	**1a**	**2a**	**3a**	**3b**
Empirical fomula	$C_{10}H_{11}BrO_3$	$C_{12}H_{13}N_3O_3$	$C_{11}H_{11}N_3O_3$	$C_{12}H_{13}N_3O_4$
Fomula weight	259.10	247.25	233.23	263.25
Temperature (K)	296(2)	296(2)	296(2)	296(2)
Wavelength (Å)	0.71073	0.71073	0.71073	0.71073
Crystal system	Monoclinic	Monoclinic	Monoclinic	Orthorhombic
Space group	$P2_1/n$	$P2_1$	$P2_1$	$Pca2_1$
a (Å)	4.7745(6)	8.8786(15)	4.5923(7)	17.8859(17)
b (Å)	18.454(3)	4.4485(8)	19.343(3)	4.0963(5)
c (Å)	12.3429(17)	15.455(3)	6.4024(10)	17.4011(17)
α (°)	90	90	90	90
β (°)	99.501(2)	107.779(2)	93.168(2)	90
γ (°)	90	90	90	90
V (Å^3)	1072.6(2)	597.58(18)	567.85(15)	1274.9(2)
Z	4	2	2	4
Dc (g cm^{-3})	1.604	1.374	1.364	1.372
μ (mm^{-1})	3.811	1.033	0.102	0.105
F(000)	520	260	244	552
Crystal size (mm)	0.18 x 0.13 x 0.12	0.20 x 0.15 x 0.13	0.21 x 0.16 x 0.13	0.23 x 0.17 x 0.14
Θ range for date	2.77-22.65	2.453-25.495	3.187-25.498	4.188-25.482
Reflections collected	8187	4646	4385	2828
Independent reflection	1996 [R(int) = 0.0274]	2210 [R(int) = 0.0181]	2096 [R(int) = 0.0154]	1730 [R(int) = 0.0181]
Goodness-of-fit on F^2	1.026	1.038	1.083	1.011
Final R indices[$I > 2\sigma(I)$]^a	R_1= 0.0323,wR_2= 0.0788	R_1= 0.0283, wR_2= 0.0742	R_1= 0.0307, wR_2= 0.0685	R_1= 0.0364, wR_2= 0.0739
R indices(all data)a	R_1=0.0497, wR_2= 0.0874	R_1= 0.0315, wR_2= 0.0766	R_1= 0.0398, wR_2= 0.0727	R_1= 0.0441, wR_2= 0.0782

$^a R_1 = \Sigma \,\|F_o| - |F_c\| / \Sigma |F_o|$; $^b wR_2 = [\Sigma w(F_o^2 - F_c^2)_2 / \Sigma w(F_o^2)^2]^{1/2}$

Table S2. Selected bond lengths (Å) and bond angles (°) of **1a**-**3a** and **3b**

**Bond**	**Bond**	**Angle**	**Bond**	**Angle**	**Bond**
			**1a**		
C(1)-O(3)	1.364(3)	O(3)-C(1)-C(2	125.0(2)	C(5)-C(6)-C(1)	120.6(3)
C(1)-C(2)	1.382(4)	O(3)-C(1)-C(6	115.4(2)	O(1)-C(7)-O(2)	122.5(3)
C(1)-C(6)	1.389(4)	C(2)-C(1)-C(6	119.6(2)	O(1)-C(7)-C(4)	124.5(3)
C(2)-C(3)	1.377(4)	C(3)-C(2)-C(1	119.5(3)	O(2)-C(7)-C(4)	113.0(2)
C(4)-C(5)	1.385(4)	C(2)-C(3)-C(4	121.3(2)	O(3)-C(9)-C(10	108.2(2)
C(3)-C(4)	1.389(4)	C(3)-C(4)-C(5	118.8(2)	C(9)-C(10)-Br(1	111.6(2)
C(4)-C(7)	1.478(4)	C(3)-C(4)-C(7	118.6(2)	C(7)-O(2)-C(8)	116.9(3)

C(5)-C(6)	1.371(4)	C(5)-C(4)-C(7	122.6(2)	C(1)-O(3)-C(9)	117.4(2)
C(7)-O(1)	1.201(3)	C(6)-C(5)-C(4	120.2(3)		
C(7)-O(2)	1.328(3)				
C(8)-O(2)	1.441(4)				
C(9)-O(3)	1.427(3)				
C(9)-C(10)	1.489(4)				
C(10)-Br(1)	1.942(3)				

2a

C(1)-C(2)	1.375(3)	C(2)-C(1)-C(6	120.57(18)	O(2)-C(7)-C(6)	112.10(17)
C(1)-C(6)	1.394(3)	C(1)-C(2)-C(3	120.22(18)	O(3)-C(9)-C(10	107.93(15)
C(2)-C(3)	1.383(3)	O(3)-C(3)-C(2	116.20(16)	N(1)-C(10)-C(9	112.61(19)
C(3)-O(3)	1.368(2)	O(3)-C(3)-C(4	123.78(17)	N(2)-C(11)-N(3	115.4(2)
C(3)-C(4)	1.389(3)	C(2)-C(3)-C(4	120.02(19)	N(3)-C(12)-N(1	111.52(18)
C(4)-C(5)	1.379(3)	C(5)-C(4)-C(3	119.09(17)	C(12)-N(1)-N(2	109.06(17)
C(5)-C(6)	1.383(3)	C(4)-C(5)-C(6	121.70(17)	C(12)-N(1)-C(1	130.05(17)
C(6)-C(7)	1.473(3)	C(5)-C(6)-C(1	118.39(18)	N(2)-N(1)-C(10	120.89(15)
C(7)-O(1)	1.202(2)	C(5)-C(6)-C(7	122.59(17)	C(11)-N(2)-N(1	102.15(16)
C(7)-O(2)	1.349(2)	C(1)-C(6)-C(7	119.02(17)	C(12)-N(3)-C(1	101.82(18)
C(8)-O(2)	1.445(3)	O(1)-C(7)-O(	122.4(2)	C(7)-O(2)-C(8)	116.23(17)
C(9)-O(3)	1.430(2)	O(1)-C(7)-C(6	125.48(18)	C(3)-O(3)-C(9)	117.43(14)
C(9)-C(10)	1.503(3)				
C(10)-N(1)	1.453(3)				
C(11)-N(2)	1.316(3)				
C(11)-N(3)	1.337(3)				
C(12)-N(3)	1.312(3)				
C(12)-N(1)	1.321(3)				
N(1)-N(2)	1.347(2)				

3a

C(1)-C(2)	1.363(4)	C(2)-C(1)-C(6	121.1(2)	O(2)-C(7)-C(6)	114.4(2)
C(1)-C(6)	1.388(4)	C(1)-C(2)-C(3	120.1(2)	O(3)-C(8)-C(9)	106.2(2)
C(2)-C(3)	1.371(3)	O(3)-C(3)-C(2	115.0(2)	N(1)-C(9)-C(8)	111.8(2)
C(3)-O(3)	1.363(3)	O(3)-C(3)-C(4	124.9(2)	N(1)-C(10)-N(3	110.7(2)
C(3)-C(4)	1.381(3)	C(2)-C(3)-C(4	120.1(3)	N(2)-C(11)-N(3	114.5(2)
C(4)-C(5)	1.374(4)	C(5)-C(4)-C(3	119.1(2)	C(10)-N(1)-N(2	109.4(2)
C(5)-C(6)	1.378(4)	C(4)-C(5)-C(6	121.6(2)	C(10)-N(1)-C(9	129.8(2)
C(6)-C(7)	1.478(4)	C(5)-C(6)-C(1	117.9(3)	N(2)-N(1)-C(9)	120.7(2)
C(7)-O(1)	1.211(3)	C(5)-C(6)-C(7	119.6(2)	C(11)-N(2)-N(1	102.7(2)
C(7)-O(2)	1.314(3)	C(1)-C(6)-C(7	122.5(2)	C(10)-N(3)-C(1	102.7(3)
C(8)-O(3)	1.418(3)	O(1)-C(7)-O(	122.2(3)	C(3)-O(3)-C(8)	118.7(2)
C(8)-C(9)	1.500(4)	O(1)-C(7)-C(6	123.4(3)		
C(9)-N(1)	1.455(3)				
C(10)-N(1)	1.310(3)				

C(10)-N(3)	1.313(4)
C(11)-N(2)	1.307(4)
C(11)-N(3)	1.334(4)
N(1)-N(2)	1.343(3)

3b

C(1)-O(1)	1.207(3)	O(1)-C(1)-O(	122.7(3)	C(7)-C(6)-C(5)	118.9(3)
C(1)-O(2)	1.324(4)	O(1)-C(1)-C(2	123.7(3)	C(6)-C(7)-C(2)	120.9(3)
C(1)-C(2)	1.479(4)	O(2)-C(1)-C(2	113.5(3)	O(3)-C(9)-C(10	106.1(3)
C(2)-C(3)	1.380(4)	C(3)-C(2)-C(7	119.6(3)	N(1)-C(10)-C(9	113.5(2)
C(2)-C(7)	1.395(4)	C(3)-C(2)-C(1	122.8(3)	C(12)-N(2)-N(1	102.4(3)
C(3)-C(4)	1.386(4)	C(7)-C(2)-C(1	117.6(3)	C(11)-N(3)-C(1	102.3(3)
C(4)-C(5)	1.374(4)	C(2)-C(3)-C(4	120.0(3)	C(11)-N(1)-N(2	109.3(3)
C(5)-O(3)	1.370(3)	C(5)-C(4)-C(3	120.3(3)	C(11)-N(1)-C(1	130.5(3)
C(5)-C(6)	1.401(4)	O(3)-C(5)-C(4	125.1(3)	N(2)-N(1)-C(10	120.2(2)
C(6)-O(4)	1.365(4)	O(3)-C(5)-C(6	114.5(3)	N(3)-C(11)-N(1	111.1(3)
C(6)-C(7)	1.374(4)	C(4)-C(5)-C(6	120.4(3)	N(2)-C(12)-N(3	115.0(3)
C(8)-O(4)	1.431(3)	O(4)-C(6)-C(7	125.3(3)	C(5)-O(3)-C(9)	117.9(2)
C(9)-O(3)	1.441(3)	O(4)-C(6)-C(5	115.7(3)	C(6)-O(4)-C(8)	117.1(2)
C(9)-C(10)	1.501(4)				
C(10)-N(1)	1.463(4)				
N(2)-C(12)	1.317(4)				
N(2)-N(1)	1.349(3)				
N(3)-C(11)	1.313(5)				
N(3)-C(12)	1.346(4)				
N(1)-C(11)	1.327(4)				

2.3.1. Crystal structure of compoud **1a**

NMR

1a

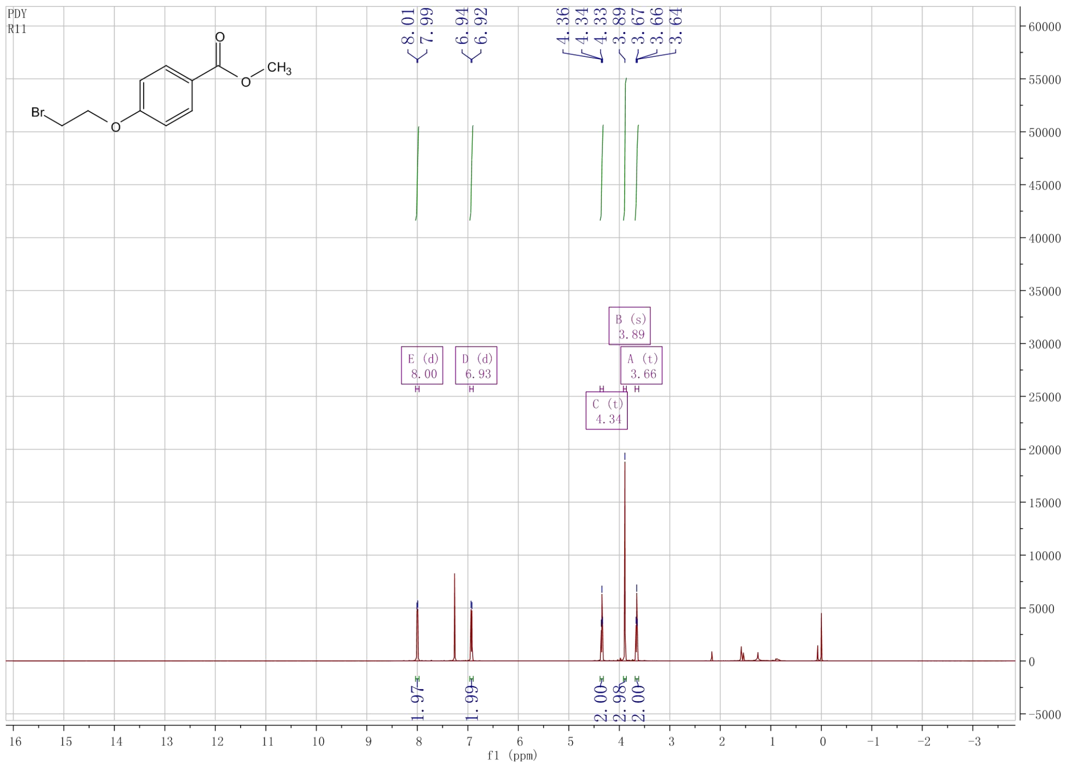
PDY
R11
Br
O
O
O
CH3
8.01
7.99
6.94
6.92
4.36
4.34
4.33
3.89
3.67
3.66
3.64
B (s)
3.89
E (d)
8.00
D (d)
6.93
A (t)
3.66
C (t)
4.34
1.97
1.99
2.00
2.98
2.00
f1 (ppm)

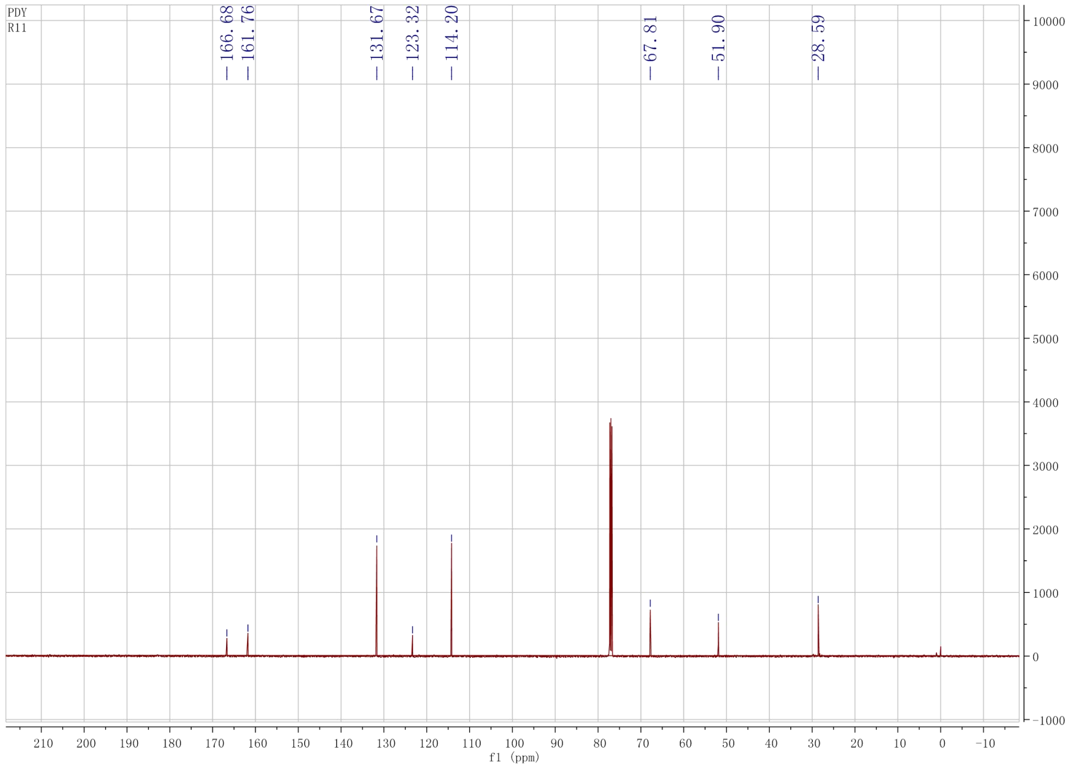
PDY
R11
166.68
161.76
131.67
123.32
114.20
67.81
51.90
28.59
f1 (ppm)

1b

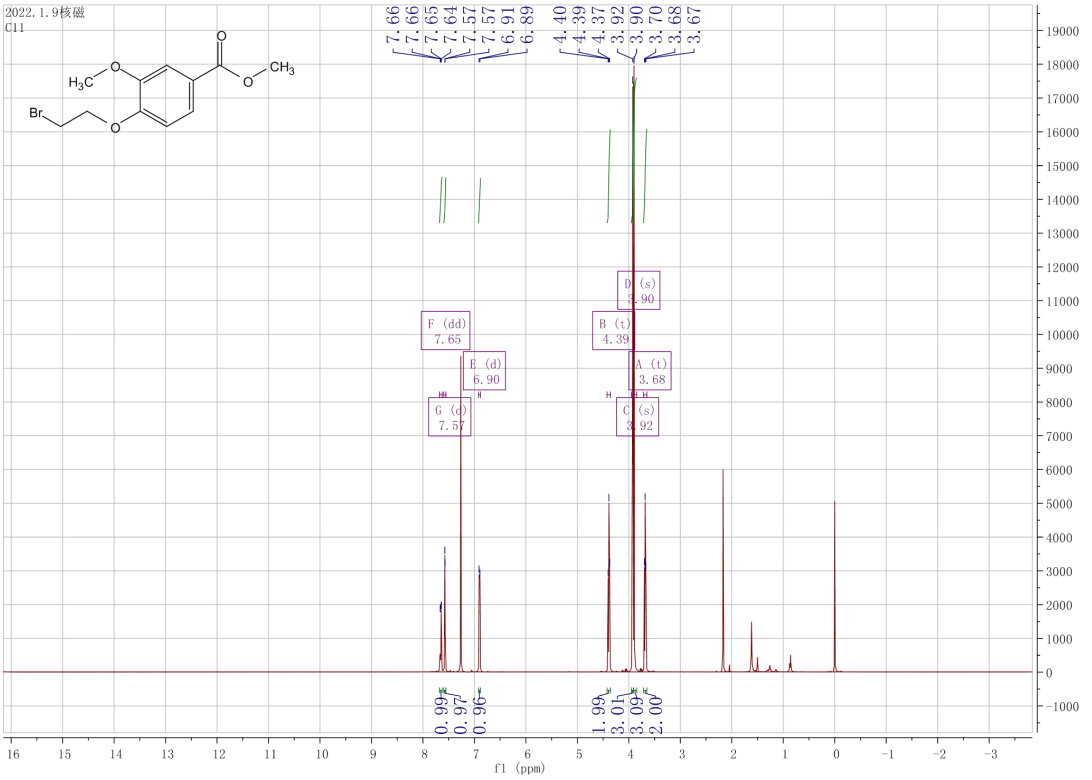
2022.1.9核磁
C11
7.66 7.66 7.65 7.64 7.57 7.57 6.91 6.89
4.40 4.39 4.37 3.92 3.90 3.70 3.68 3.67
F (dd) 7.65
E (d) 6.90
G (d) 7.57
B (t) 4.39
D (s) 3.90
A (t) 3.68
C (s) 3.92
0.99 0.97 0.96
1.99 3.01 3.09 2.00
f1 (ppm)

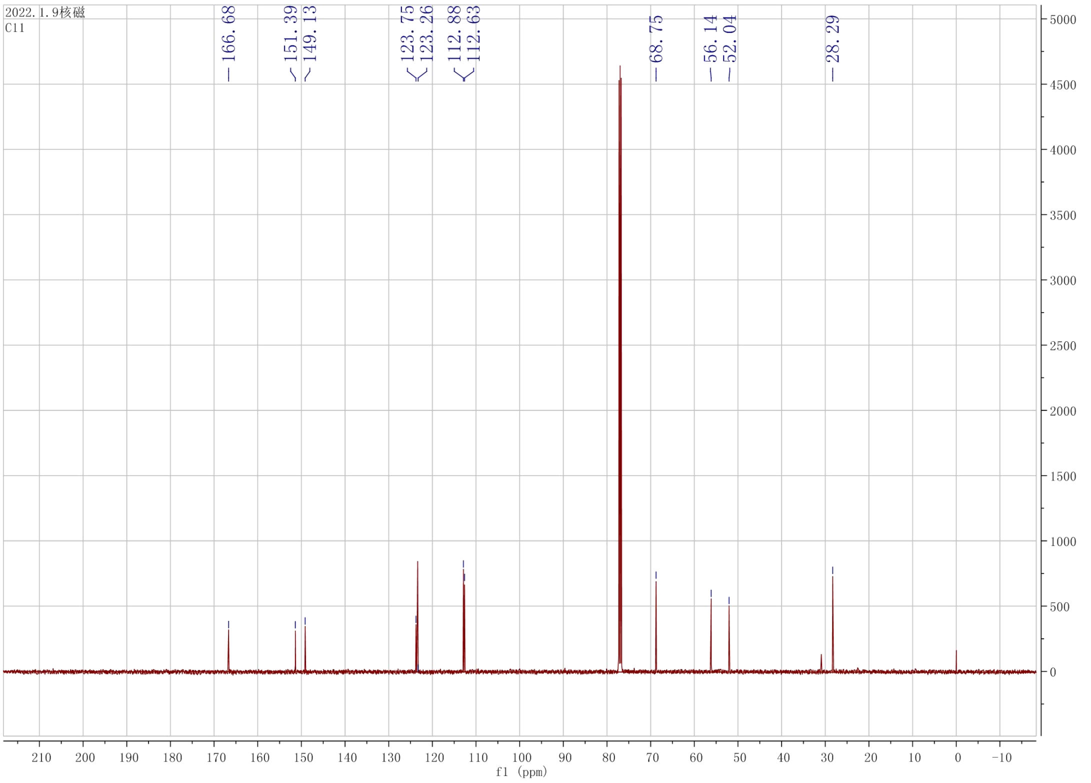
2022.1.9核磁
C11
166.68
151.39 149.13
123.75 123.26
112.88 112.63
68.75
56.14 52.04
28.29
f1 (ppm)

1c

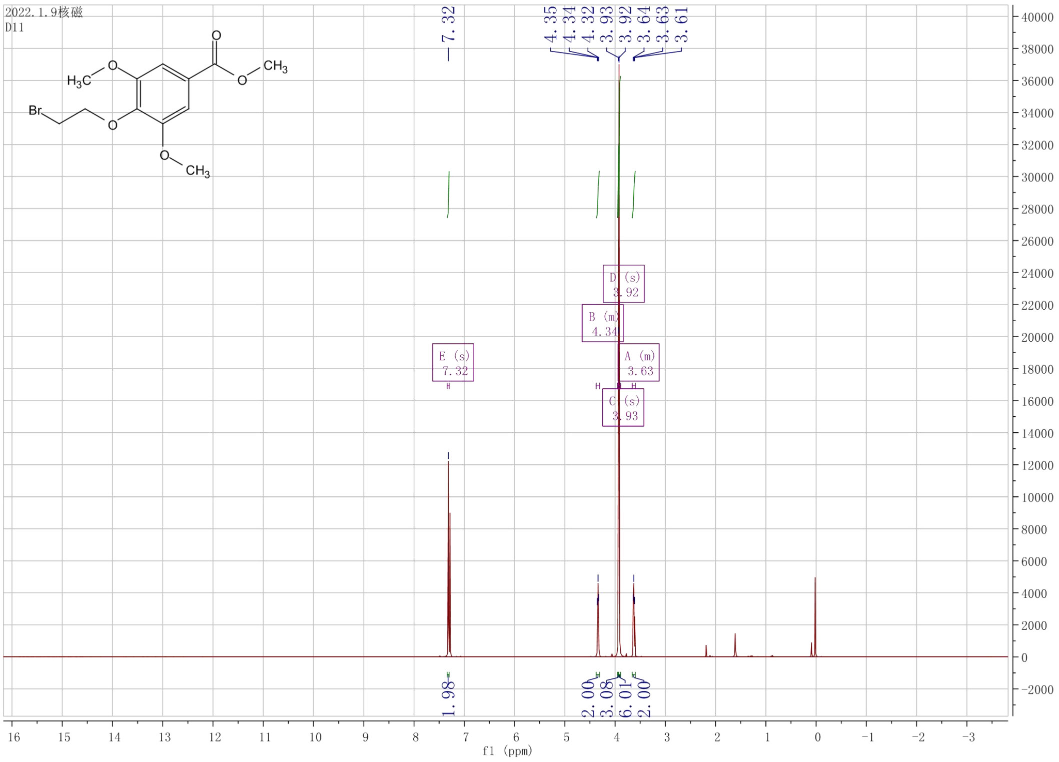

2022.1.9核磁
D11
7.32
4.35
4.34
4.32
3.93
3.92
3.64
3.63
3.61
D (s)
3.92
B (m)
4.34
E (s)
7.32
A (m)
3.63
C (s)
3.93
1.98
2.00
3.08
6.01
2.00
f1 (ppm)

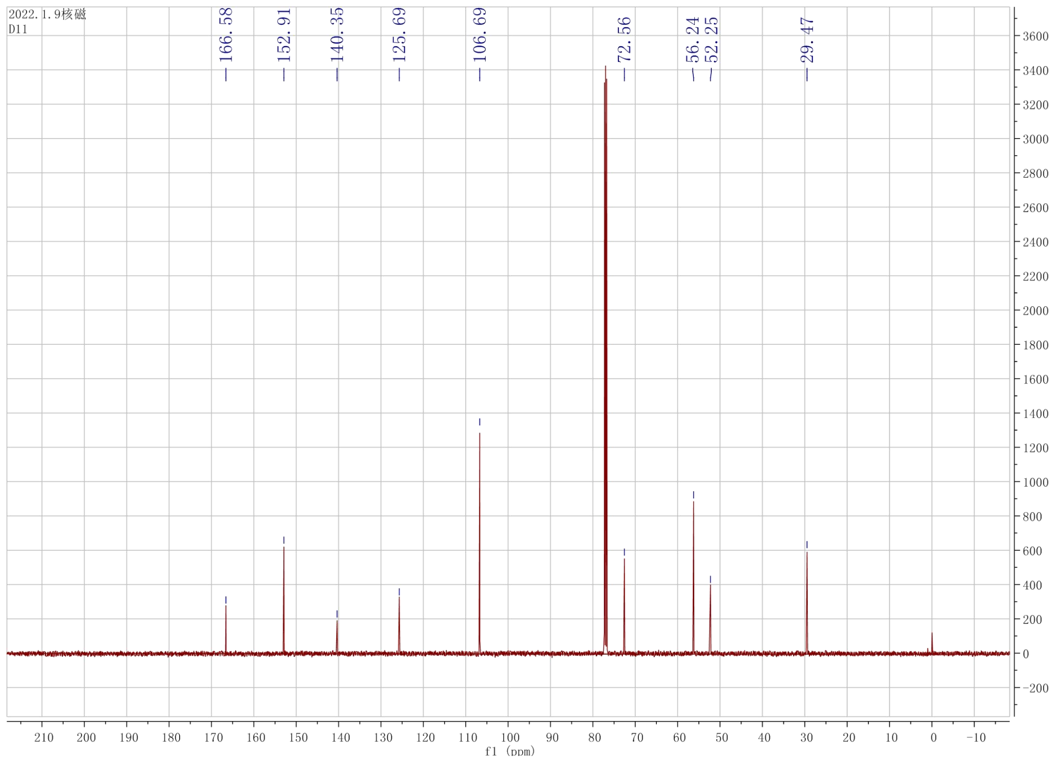
2022.1.9核磁
D11
166.58
152.91
140.35
125.69
106.69
72.56
56.24
52.25
29.47
f1 (ppm)

2a

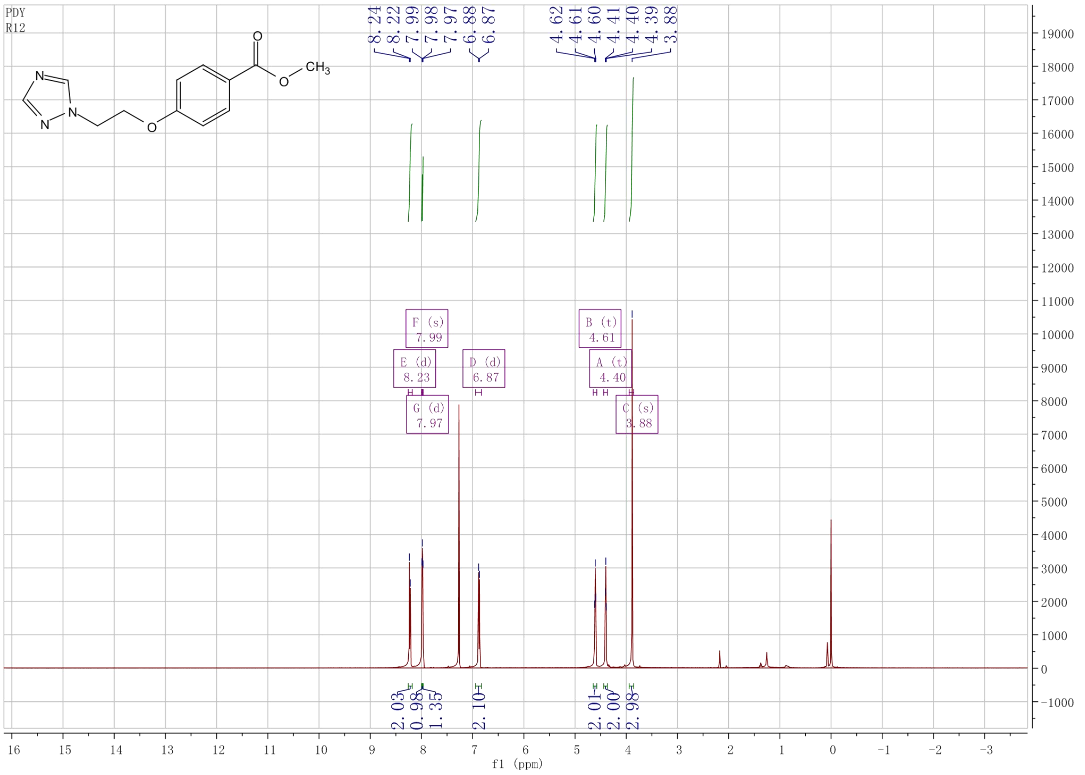
PDY
R12
8.24
8.22
7.99
7.98
7.97
6.88
6.87
4.62
4.61
4.60
4.41
4.40
4.39
3.88
F (s)
7.99
E (d)
8.23
G (d)
7.97
D (d)
6.87
B (t)
4.61
A (t)
4.40
C (s)
3.88
2.03
0.98
1.35
2.10
2.01
2.00
2.98
f1 (ppm)

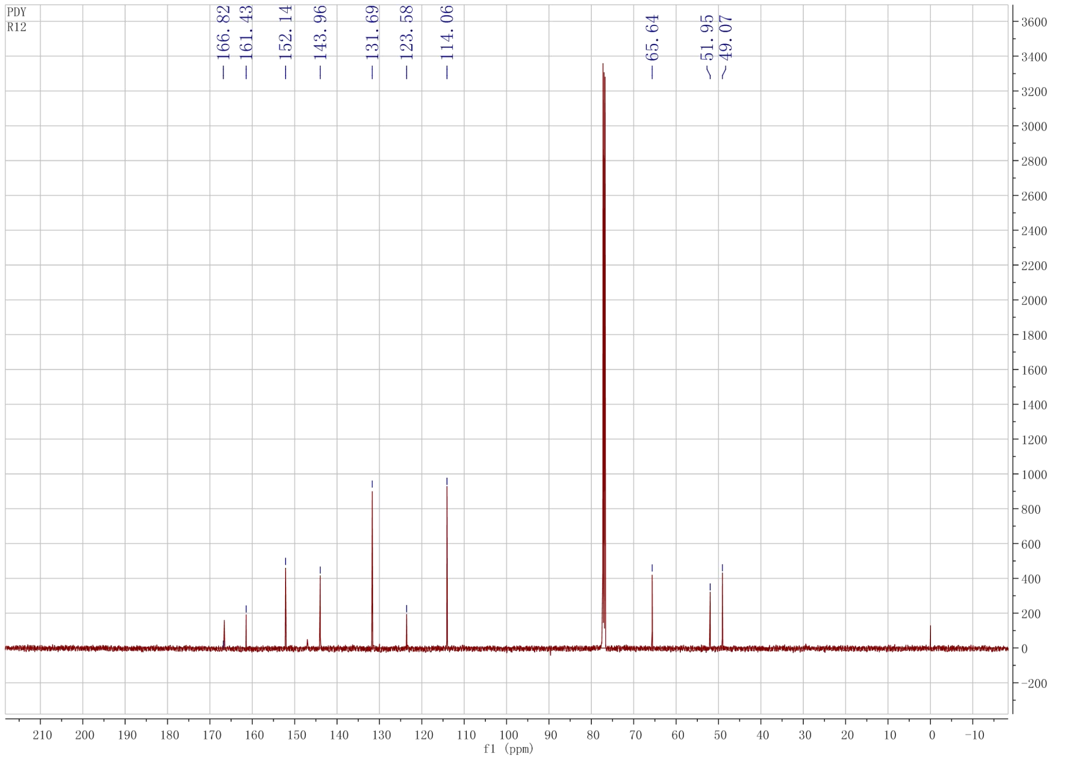
PDY
R12
166.82
161.43
152.14
143.96
131.69
123.58
114.06
65.64
51.95
49.07
f1 (ppm)

2b

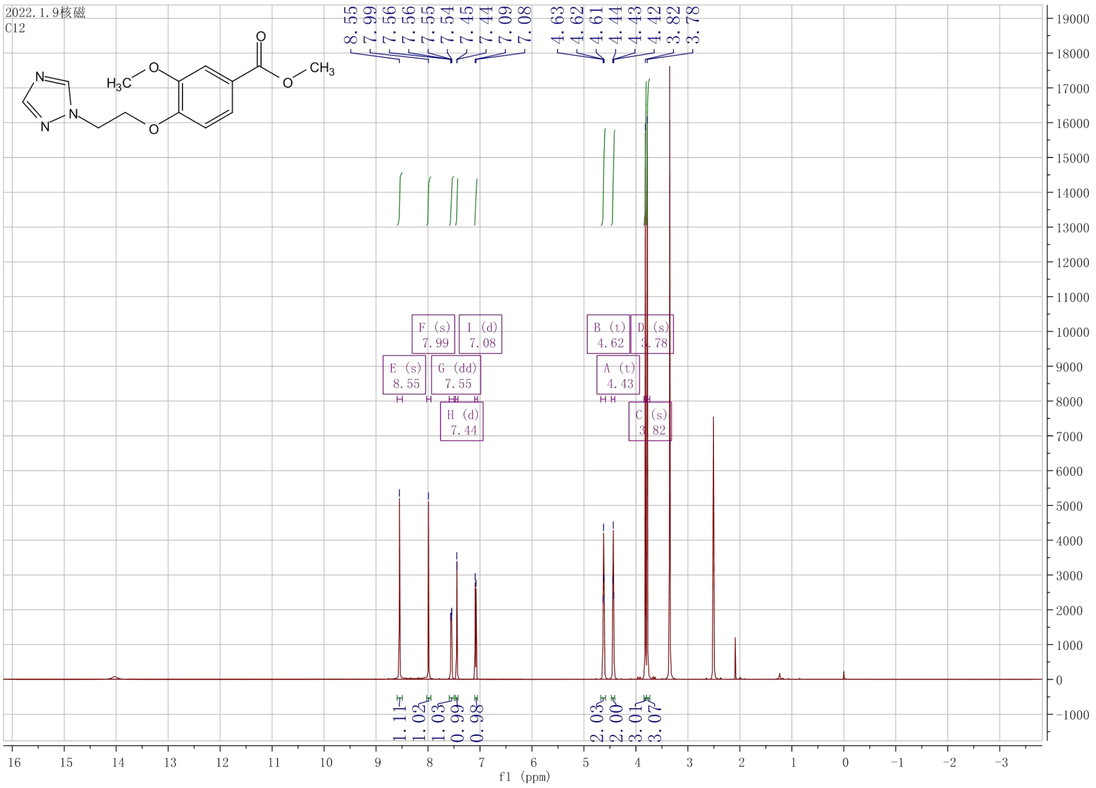
2022.1.9核磁
C12
8.55
7.99
7.56
7.56
7.55
7.54
7.45
7.44
7.09
7.08
4.63
4.62
4.61
4.44
4.43
4.42
3.82
3.78
F (s) 7.99
I (d) 7.08
E (s) 8.55
G (dd) 7.55
H (d) 7.44
B (t) 4.62
D (s) 3.78
A (t) 4.43
C (s) 3.82
1.11
1.02
1.03
0.99
0.98
2.03
2.00
3.01
3.07
f1 (ppm)

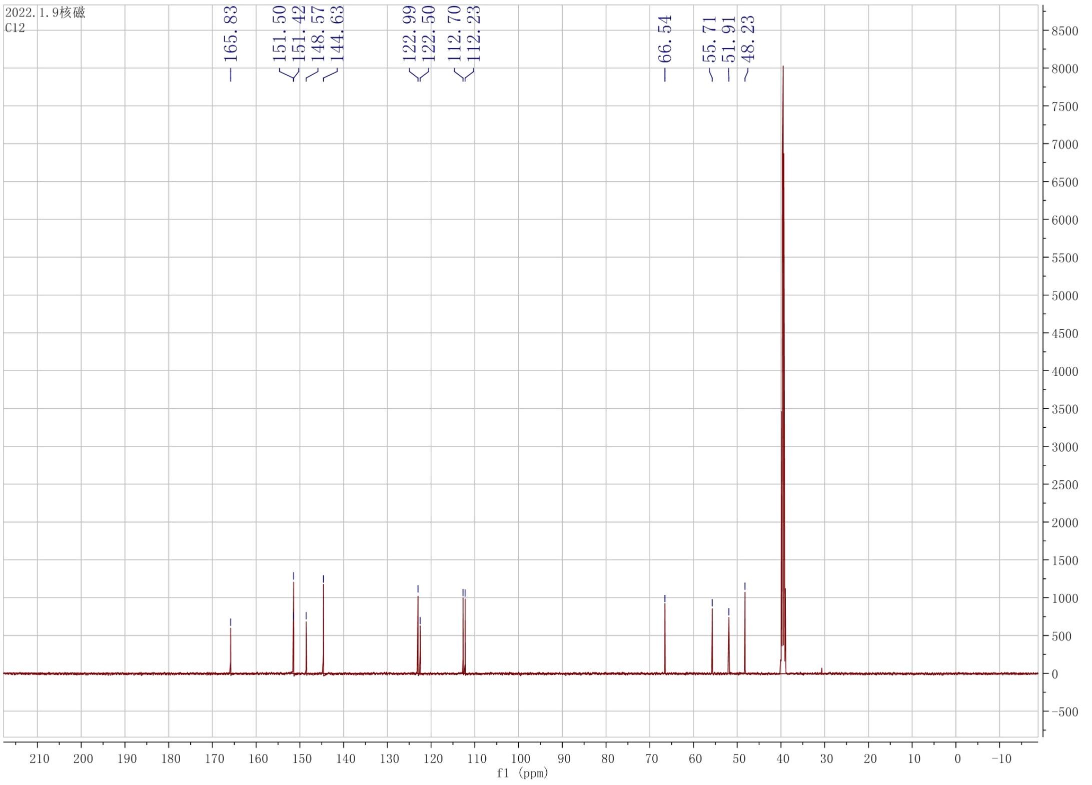
2022.1.9核磁
C12
165.83
151.50
151.42
148.57
144.63
122.99
122.50
112.70
112.23
66.54
55.71
51.91
48.23
f1 (ppm)

2c

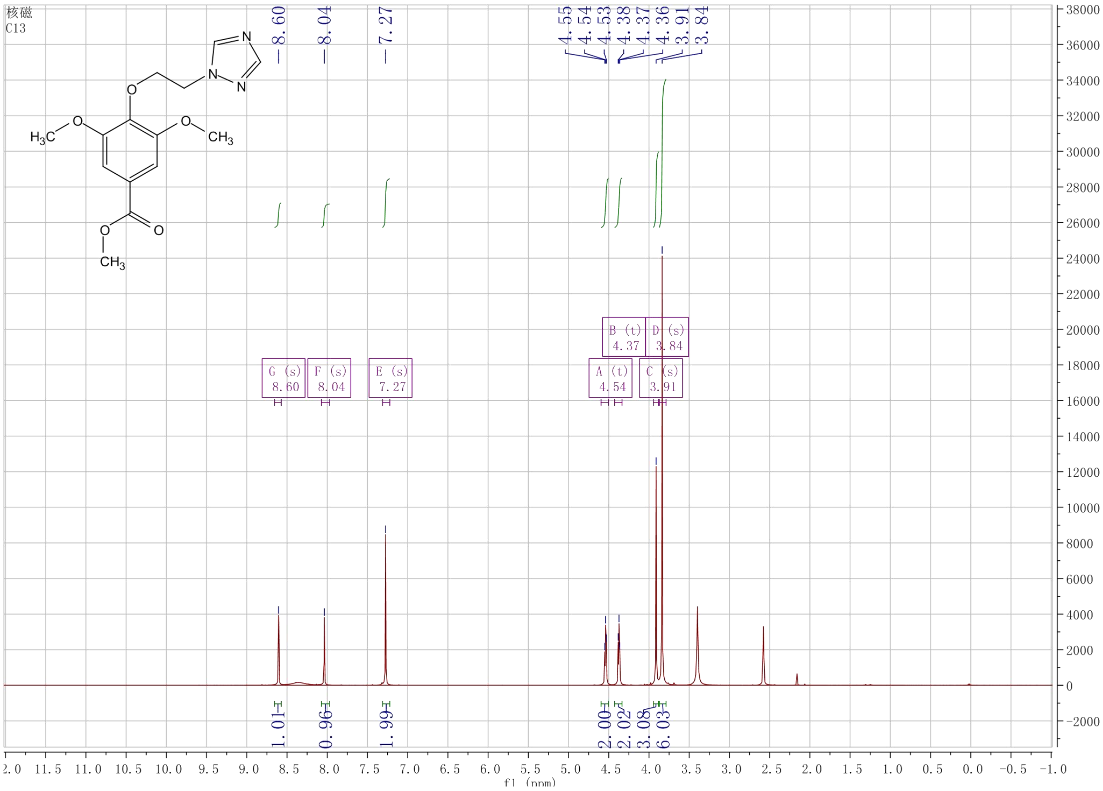

核磁
C13
8.60
8.04
7.27
4.55
4.54
4.53
4.38
4.37
4.36
3.91
3.84
G (s) 8.60
F (s) 8.04
E (s) 7.27
A (t) 4.54
B (t) 4.37
C (s) 3.91
D (s) 3.84
1.01
0.96
1.99
2.00
2.02
3.08
6.03
f1 (ppm)

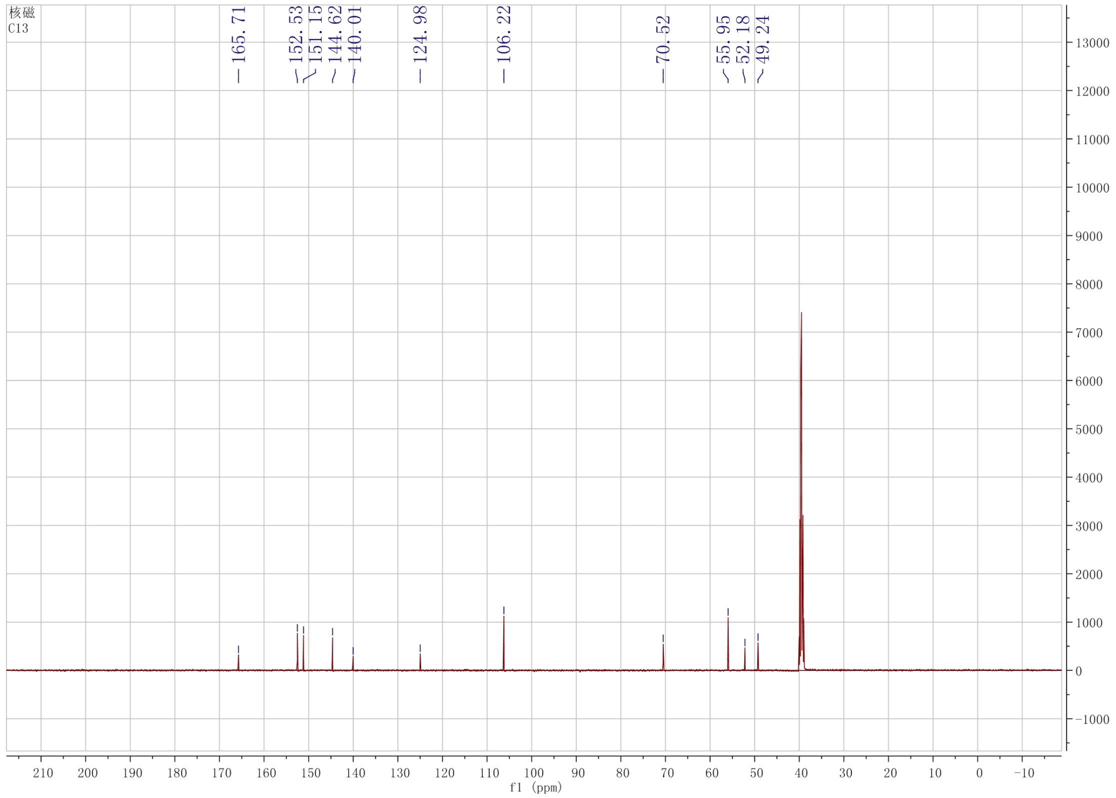

核磁
C13
165.71
152.53
151.15
144.62
140.01
124.98
106.22
70.52
55.95
52.18
49.24
f1 (ppm)

3a

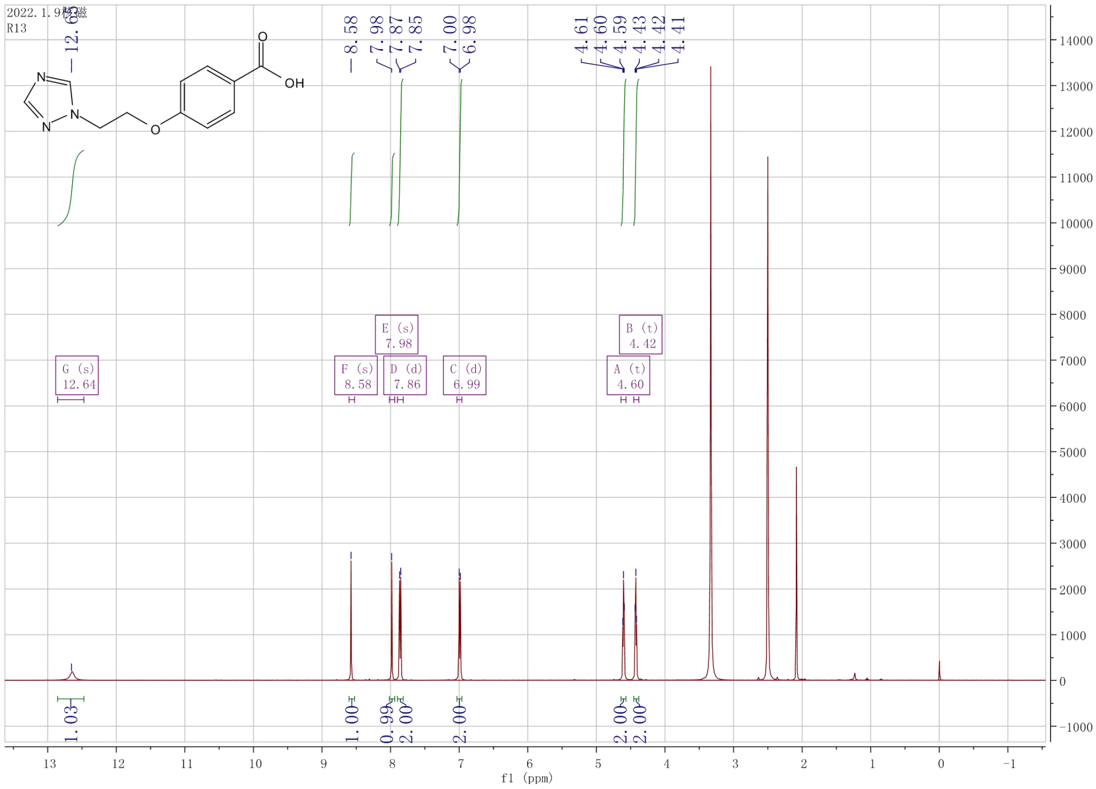
2022.1.9核磁
R13
12.65
8.58
7.98
7.87
7.85
7.00
6.98
4.61
4.60
4.59
4.43
4.42
4.41
N
N
N
O
O
OH
G (s) 12.64
F (s) 8.58
E (s) 7.98
D (d) 7.86
C (d) 6.99
A (t) 4.60
B (t) 4.42
1.03
1.00
0.99
2.00
2.00
2.00
2.00
f1 (ppm)

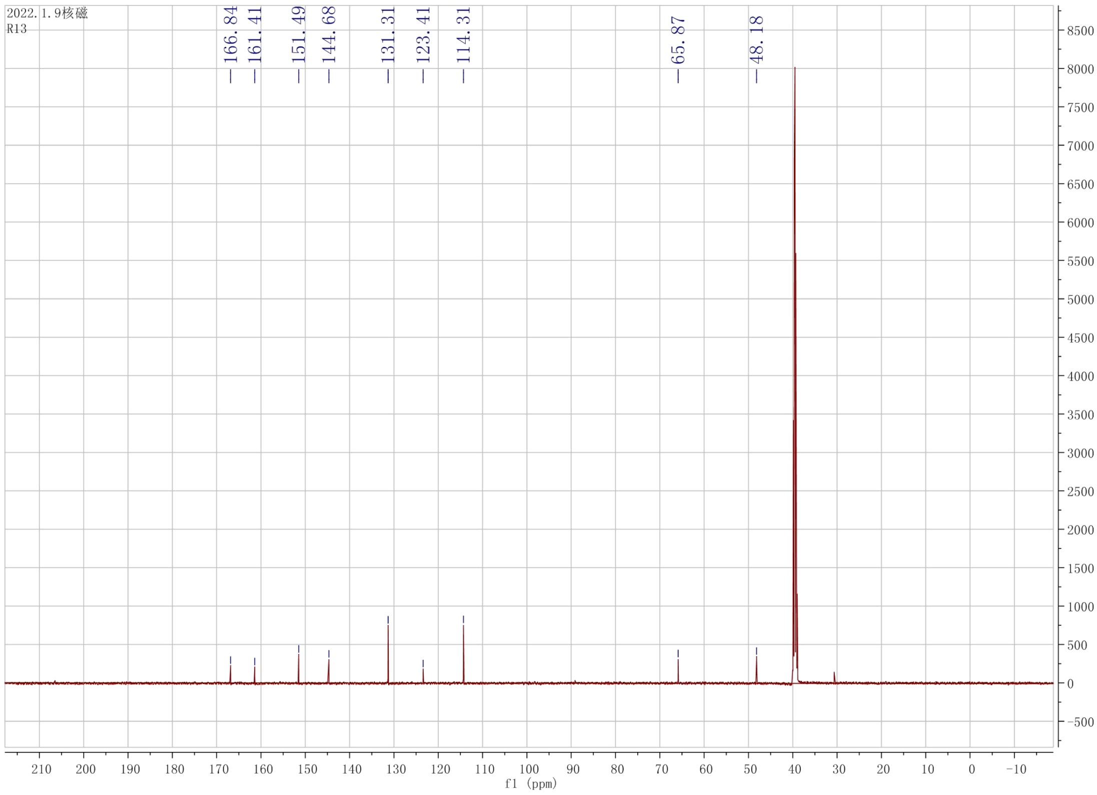
2022.1.9核磁
R13
166.84
161.41
151.49
144.68
131.31
123.41
114.31
65.87
48.18
f1 (ppm)

3b

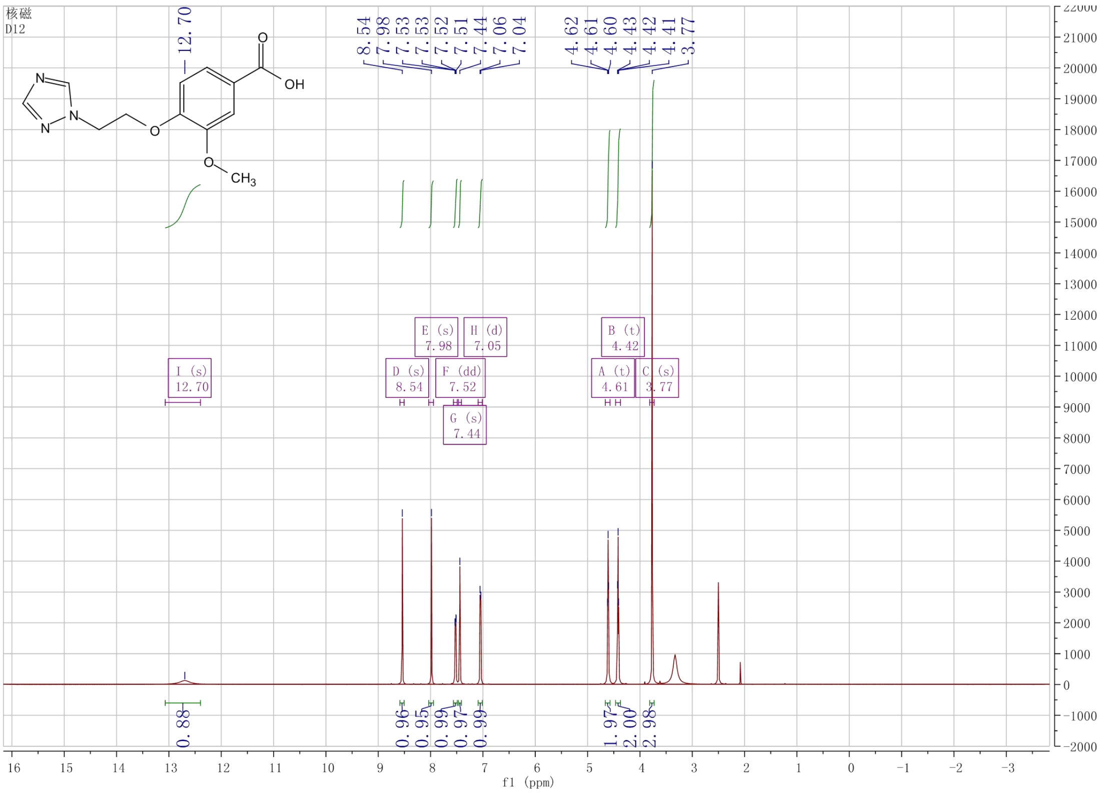

核磁
D12
12.70
8.54
7.98
7.53
7.53
7.52
7.51
7.44
7.06
7.04
4.62
4.61
4.60
4.43
4.42
4.41
3.77
OH
O
N
N
N
O
O
CH3
E (s) 7.98
H (d) 7.05
B (t) 4.42
I (s) 12.70
D (s) 8.54
F (dd) 7.52
A (t) 4.61
C (s) 3.77
G (s) 7.44
0.88
0.96
0.95
0.99
0.97
0.99
1.97
2.00
2.98
f1 (ppm)

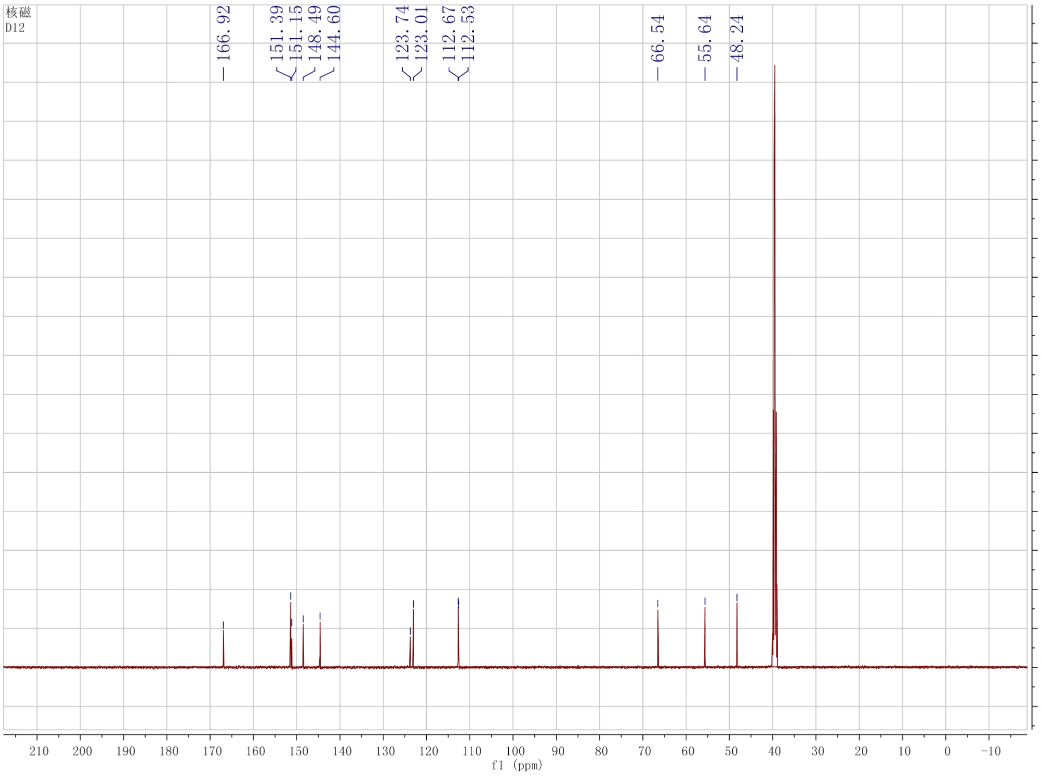
核磁
D12
166.92
151.39
151.15
148.49
144.60
123.74
123.01
112.67
112.53
66.54
55.64
48.24
f1 (ppm)

3c

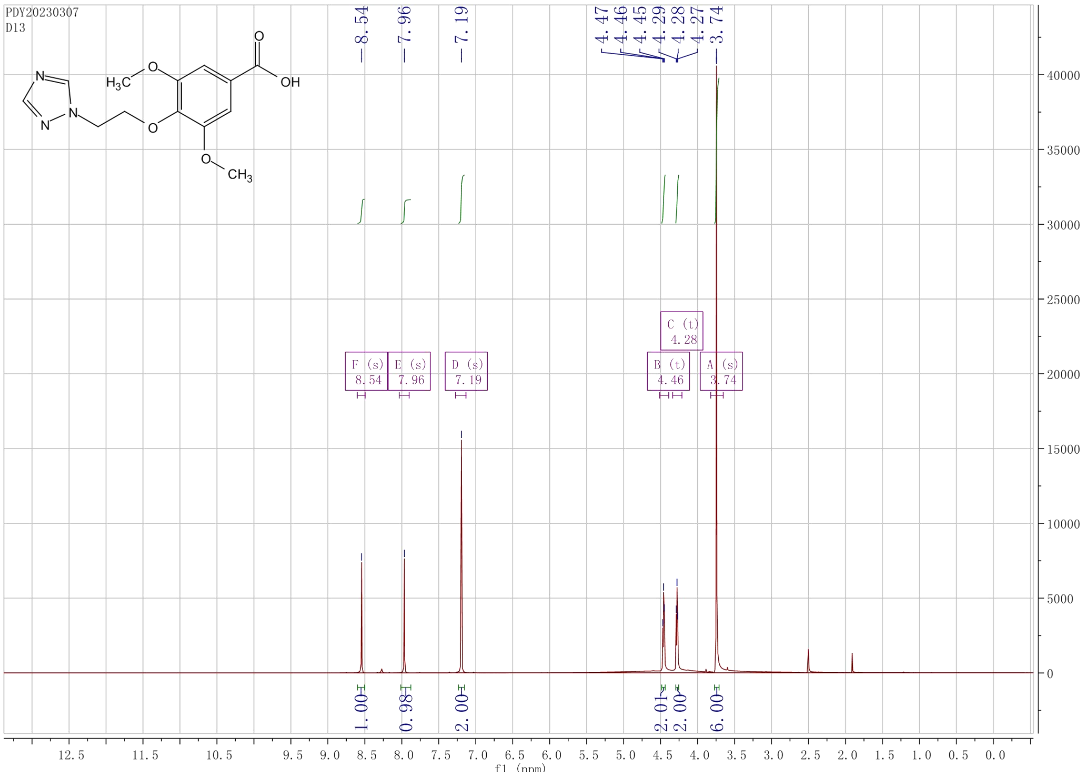
PDY20230307
D13
8.54
7.96
7.19
4.47
4.46
4.45
4.29
4.28
4.27
3.74
F (s) 8.54
E (s) 7.96
D (s) 7.19
C (t) 4.28
B (t) 4.46
A (s) 3.74
1.00
0.98
2.00
2.01
2.00
6.00
f1 (ppm)

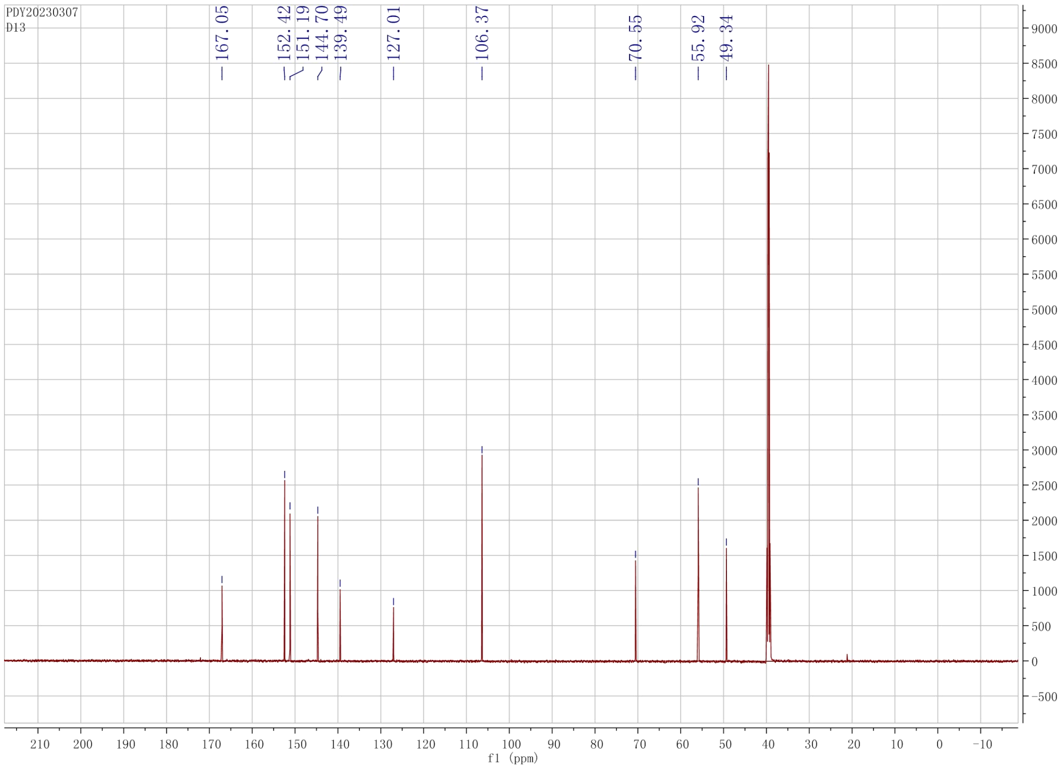
PDY20230307
D13
167.05
152.42
151.19
144.70
139.49
127.01
106.37
70.55
55.92
49.34
f1 (ppm)

HRMS

The mass spectrum of 1a:

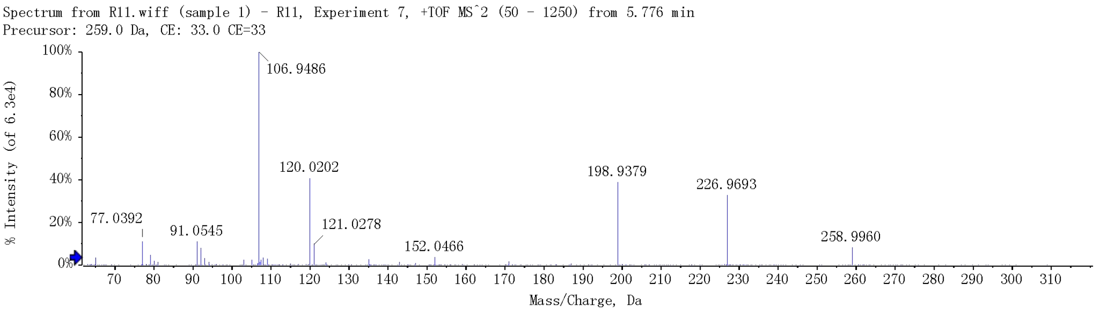

The mass spectrum of 1b:

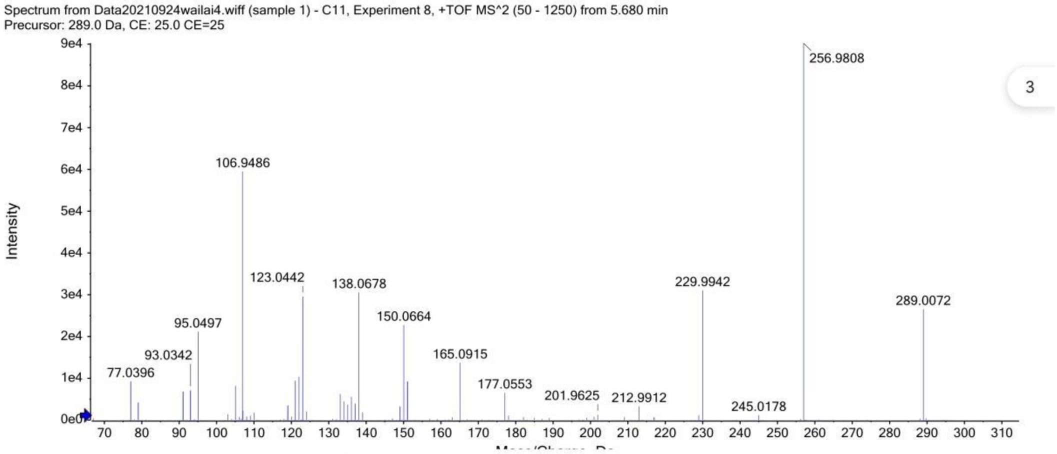

The mass spectrum of 1c:

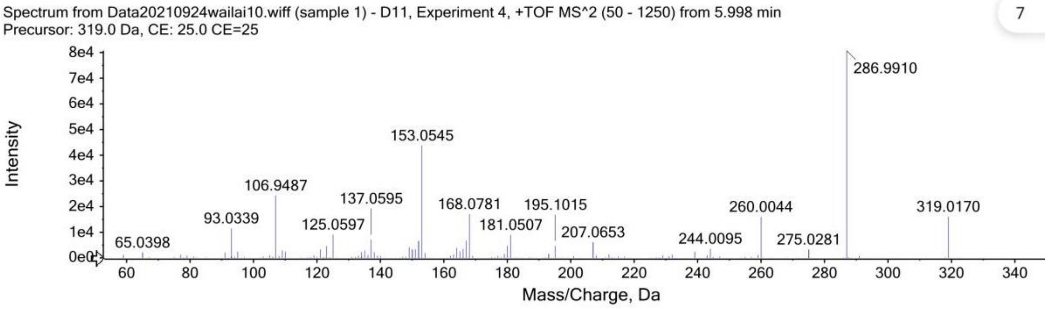

The mass spectrum of 2a:

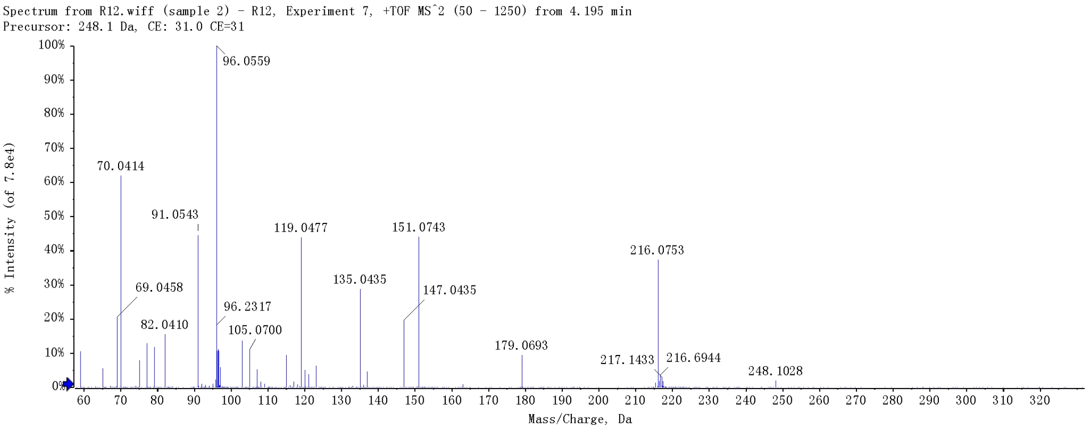

The mass spectrum of 2b:

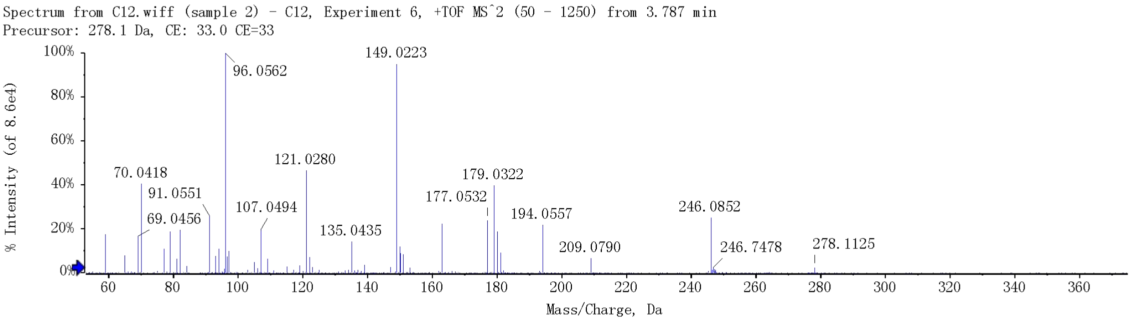

The mass spectrum of 2c:

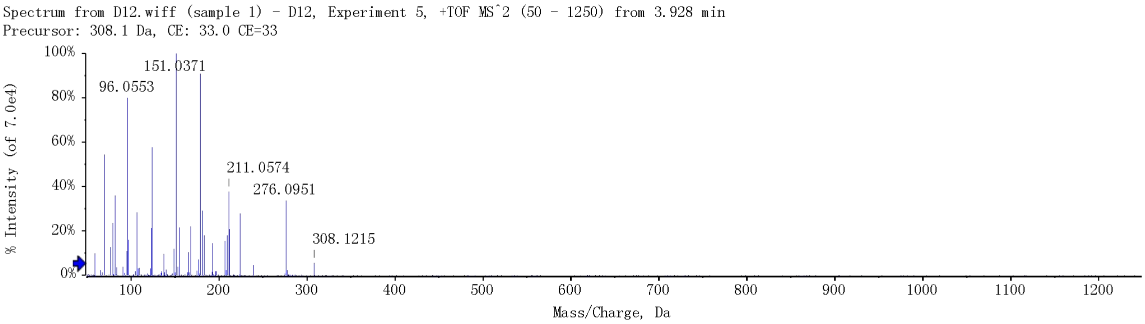

The mass spectrum of 3a:

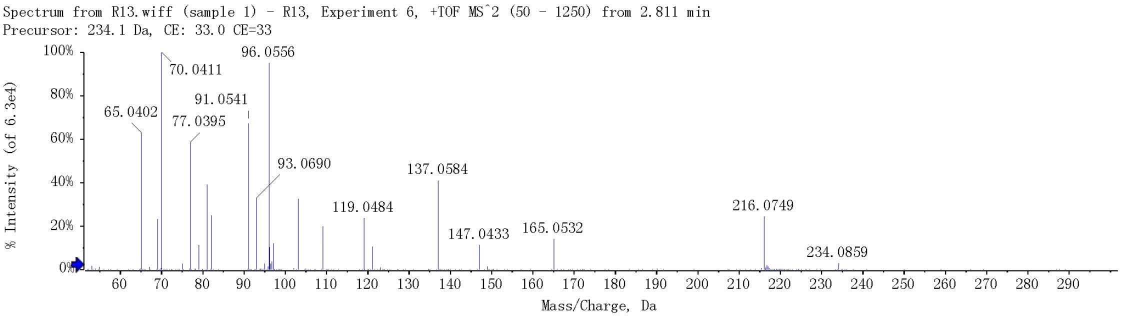

The mass spectrum of 3b:

Spectrum from C13.wiff (sample 1) - C13, Experiment 3, +TOF MS^2 (50 - 1250) from 2.806 min
Precursor: 264.1 Da, CE: 33.0 CE=33

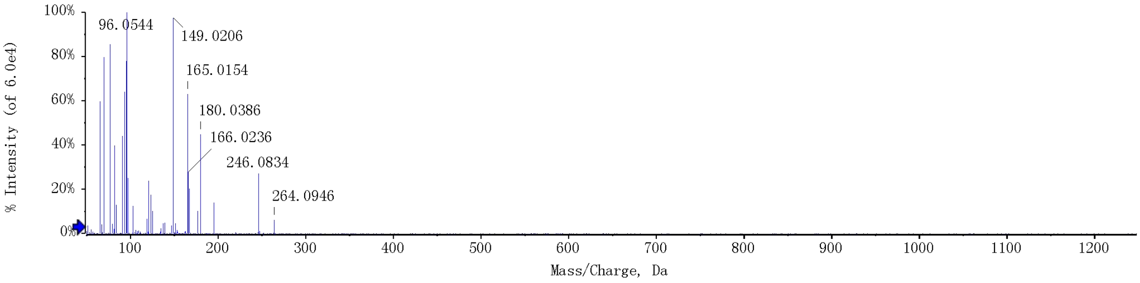

The mass spectrum of 3c:

Spectrum from D13.wiff (sample 1) - D13, Experiment 4, +TOF MS^2 (50 - 1250) from 3.030 min
Precursor: 294.1 Da, CE: 33.0 CE=33

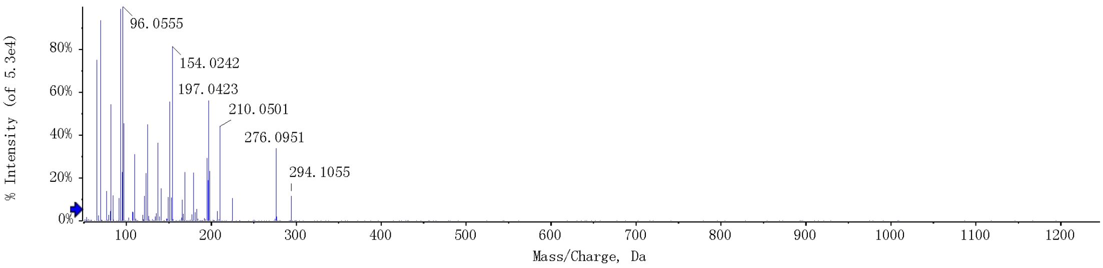

Supplement: Supplementary file 1 [file molecules-28-06970-s001.zip › molecules-2566989-supplementary.pdf]
